# A Comprehensive Overview of Medical Error in Hospitals Using Incident-Reporting Systems, Patient Complaints and Chart Review of Inpatient Deaths

**DOI:** 10.1371/journal.pone.0031125

**Published:** 2012-02-16

**Authors:** Jeantine M. de Feijter, Willem S. de Grave, Arno M. Muijtjens, Albert J. J. A. Scherpbier, Richard P. Koopmans

**Affiliations:** 1 Department of Educational Development and Research, Faculty of Health Medicine and Life Sciences, Maastricht University, Maastricht, The Netherlands; 2 Faculty of Health Medicine and Life Sciences, Institute for Medical Education, Maastricht University, Maastricht, The Netherlands; 3 Department of Internal Medicine, Maastricht University Medical Centre, Maastricht, The Netherlands; University of Maryland, School of Medicine, United States of America

## Abstract

**Background:**

Incident reporting systems (IRS) are used to identify medical errors in order to learn from mistakes and improve patient safety in hospitals. However, IRS contain only a small fraction of occurring incidents. A more comprehensive overview of medical error in hospitals may be obtained by combining information from multiple sources. The WHO has developed the International Classification for Patient Safety (ICPS) in order to enable comparison of incident reports from different sources and institutions.

**Methods:**

The aim of this paper was to provide a more comprehensive overview of medical error in hospitals using a combination of different information sources. Incident reports collected from IRS, patient complaints and retrospective chart review in an academic acute care hospital were classified using the ICPS. The main outcome measures were distribution of incidents over the thirteen categories of the ICPS classifier “Incident type”, described as odds ratios (OR) and proportional similarity indices (PSI).

**Results:**

A total of 1012 incidents resulted in 1282 classified items. Large differences between data from IRS and patient complaints (PSI = 0.32) and from IRS and retrospective chart review (PSI = 0.31) were mainly attributable to behaviour (OR = 6.08), clinical administration (OR = 5.14), clinical process (OR = 6.73) and resources (OR = 2.06).

**Conclusions:**

IRS do not capture all incidents in hospitals and should be combined with complementary information about diagnostic error and delayed treatment from patient complaints and retrospective chart review. Since incidents that are not recorded in IRS do not lead to remedial and preventive action in response to IRS reports, healthcare centres that have access to different incident detection methods should harness information from all sources to improve patient safety.

## Introduction

It has been increasingly recognised that hospitals can be dangerous places for patients, since medical errors have been shown to cause harm to patients [1], [2]. In order to identify medical errors, to learn from mistakes and to improve patient safety, the healthcare community has introduced incident reporting systems (IRS) [2]. Since 2008, Dutch hospitals are required by law to have in place a safety management system, and with that an IRS. Studies have shown that IRS can have a positive effect on the safety climate of a hospital [3] and afford a global overview of incidents. However, as IRS are based on voluntary reporting a non-punitive environment has to be present in hospitals to generate high reporting rates [2]. That this type of environment is difficult to achieve may be reflected in the fact that IRS have been reported to reveal only the tip of the iceberg of incidents [2], [4], [5], estimated at 10% at most [6]. It is not known whether the reported 10% is representative of all errors. However, if IRS do not capture all types of error and if action to address errors is based on IRS reports, patient safety may not be served optimally.

IRS are not the only source of information for studies of incidents in hospitals. Thomas et al. described eight methods of detecting errors and adverse events, including chart review, malpractice claims analysis, observation of patient care, and IRS [7]. Although retrospective chart review is considered the gold standard [2], [8], and has been used in many studies [1], [9], [10], all methods have strengths and weaknesses, with some focusing specifically on latent (system) errors and others on active errors. [7], [11] Most studies of patient safety and adverse events rely on healthcare workers for information, but patients have also been shown to be a useful source of information, which should not be ignored [12]–[17]. A combination of information about adverse events from patients and healthcare workers may thus offer a more comprehensive representation of hospital incidents. Other studies have used such a combination, which yielded a more comprehensive picture of adverse events [14], [18]–[21]. However, the optimal combination of detection methods, where the weaknesses of one method can be overcome by another method, remains to be ascertained.

In the absence of a universally accepted incident classification system, studies focusing on IRS have used different classification systems [9], [10], [22]–[25], which hampers comparisons across studies and univocal conclusions. In an attempt to define a standardised set of patient safety concepts, the World Health Organisation (WHO) recently developed the International Classification of Patient Safety (ICPS) [26]–[28]. Although ICPS is still being tested and some criticism has been voiced [29], it appears to be an important step towards a comprehensive overview of concepts related to patient safety which, if proven successful, can facilitate comparison of results from different information sources both within and between institutions, on a local as well as national and international level.

For the present study we retrieved information from different sources (incident reports, patient complaints and retrospective chart review of deceased patients) to identify incidents and adverse events. We classified this combined information using the ICPS in order to create a comprehensive picture of incidents occurring in hospitals. We specifically addressed the following research question: Are the different information sources complementary with regard to the types of incidents they report?

## Methods

### Ethical considerations

In accordance with Dutch Law on Medical Scientific Research, retrospective research using patient charts was automatically granted ethics approval in the participating institutions and there was no requirement for individual patient consent, provided confidentiality was maintained.

### Setting

We collected data from a medium sized (700 beds) academic acute care hospital in the Netherlands, serving both adults and paediatric patients. Three information sources were used: 1) all incident reports for 2007; 2) patient complaints filed in 2007; 3) retrospective chart reviews of all inpatients that died in 2008. These information sources applied to different subgroups able to provide information about adverse events in hospitals. We used data for 2007 to ensure that incidents could not be traced back to staff or patients and referred to events before the introduction of statutory safety management systems in 2008.

### Information Sources

Because of anonymity of patient and staff information, overlap between incidents from different sources could not be detected. The results are therefore not presented as absolute differences between information sources, but as distributions of incidents over categories.

#### Incident reports

Incidents were reported on paper. All hospital personnel are authorised to report incidents, and the IRS contains information about nature, severity and place of incidents and about action taken to prevent recurrence. We transformed the available data for 2007 into a digital data file.

#### Patient complaints

Any patient can file a complaint against the hospital or an individual healthcare provider. We collected all written patient complaints, handled in 2007 by a complaint mediator or the complaints committee. Complaints not directly related to patient care (such as complaints about billing) were not included in the study (N = 59).

#### Retrospective chart review of all deceased patients

The hospital has a committee, consisting of six medical doctors and seven nurses, which retrospectively inspects the files of all deceased patients in order to identify any adverse events. The review method and definitions are based on similar national research [30], in which an adverse event is defined as “an unintended (physical and /or mental) injury which resulted in temporary or permanent disability, death or prolongation of hospital stay, and was caused by healthcare management rather than the patient's disease” [9]. All files are scanned by trained nurses, looking for triggers suggesting the occurrence of an adverse event. If an event is suspected, a medical doctor scrutinises the file to determine whether an event actually occurred and whether it was avoidable [30]. If an error is identified, the file is submitted to the full committee of medical doctors who discuss the case to reach consensus as to whether the event could have been avoided. The attending physician of the patient in question is always involved in this process. He or she comments on the committee's preliminary judgements and is notified of the final outcome of the procedure. For 119 out of 744 files of deceased patients the committee requested additional information from the attending physician. Avoidable adverse events were identified in the files of 44 patients (5.9%). Similar numbers percentages have been reported in other national research [9].

We use the term ‘reports’ to refer to incidents from the IRS, from patient complaints and from retrospective chart review.

### International Classification of Patient Safety (ICPS)

We classified all reports as ‘incident type’. This ICPS classifier, which contains thirteen categories (table 1), each with subcategories [26], [31], was deemed to be most suitable to our data.

**Table 1:** Overview of all categories in the classifier “Incident Type” (adapted from ICPS), with examples to clarify each category.

	Category	Example
**1**	behaviour	treatment of patient by staff was inconsiderate or rude
**2**	blood/ blood products	request for a blood product was for the wrong patient; or blood with the wrong blood type was administered to a patient
**3**	clinical administration	wrong documents were filled out for admission; or a patient was treated by different doctor than previously discussed
**4**	clinical process/ procedure	a delay in treatment due to postponement of surgery; or a diagnosis was missed
**5**	documentation	patient chart was missing; or information on patient chart was incorrect or missing
**6**	health care ass. infection	patient develops infection near the surgical site, due to a gauze that has been left behind in the wound.
**7**	infrastructure	trolley does not fit into the lift; or nurse slips on wet floor
**8**	medical device/ equipment	computer malfunction or surgical tools that break or are unsterile
**9**	medication/iv fluids	wrong drug is administered to the patient; or patient has not received medication
**10**	nutrition	wrong quantity or wrong sort of drip-feed is administered
**11**	oxygen/gas/vapour	patient returns from procedure and a nurse forgets to connect the oxygen
**12**	patient accidents	patient that has fallen out of bed; or patient that has fallen in the bathroom
**13**	resources/organizational management	understaffing or no available beds

A report can fall into several categories [26]. The maximum in this study was three (box S1). As we aimed to identify different types of incidents, all categories deemed pertinent to a report were included in the analysis, which resulted in a total of 1282 classified items.

#### Procedure

JMF classified a sample (from all three sources) of 300 reports and discussed the results with a second researcher (RPK) until consensus was reached. JMF then classified the remaining reports, while a random sample of 10% was also classified by RPK in order to determine interrater reliability using Cohen's kappa. Since Cohen's kappa is based on the assumption that one item cannot be in more than one category, only the first classification of each report, representing the main category for that report, was used to calculate kappa. Kappa was 0.73, indicating substantial interrater agreement [32].

#### Suitability of the ICPS

There are several reasons why we deemed the ICPS suitable for our study: 1) It was developed using a Delphi procedure [28] among stakeholders from different fields, which ensures a broad view of patient safety; 2) A standardised classification, like the ICPS, enables comparison and replication of results within and between institutions and studies; 3) The classifier ‘Incident Type’ enabled us to start with a global classification in categories, followed by a more specific classification in subcategories, thereby creating a detailed classification of each report; 4) The ICPS discriminated distinctively between the thirteen categories, which made it possible to use all of them; 5) All incidents fell into one of the thirteen categories, thus no categories were lacking in the ICPS.

### Statistical Analysis

We calculated the proportional similarity index (PSI) for distributions of the relative frequencies of incident reports from two information sources over ICPS categories in order to determine whether the sources were complementary [33]. The PSI ranges from 1-0, i.e. from highest possible similarity between two distributions to completely different patterns [34]. First we determined the distribution over all categories for each of the three databases. Using the IRS reports as the starting point, we compared the distributions of reports from the other two databases with the distribution of IRS reports.

We calculated odds ratios to determine if a specific ICPS category was more likely to be present in the IRS or in one of the two other information sources. A high odds ratio (OR≥2) indicates that an incident of this category is more frequently represented in either of the other information sources (patient complaints or retrospective chart review) than in the IRS. A low odds ratio (OR≤1) shows that an incident of this category was more strongly represented in the IRS. SPSS 15.0 was used for all calculations.

## Results

The number of reports from each information source and the total number of classified items (including 2^nd^ and 3^rd^ categories for some incidents) are displayed in table 2. All calculations were made for the total number of classified items (N = 1282).

**Table 2:** Overview of collected data.

Information source	Number of incidents (N)	Total number of classified items (incl. 2^nd^ and 3^rd^ category) (N)
Incident reports	736	904
Patient complaints	235	327
Retrospective chart review	44	51
Total	1015	1282

### Incident reports vs. patient complaints

**Figure 1:**
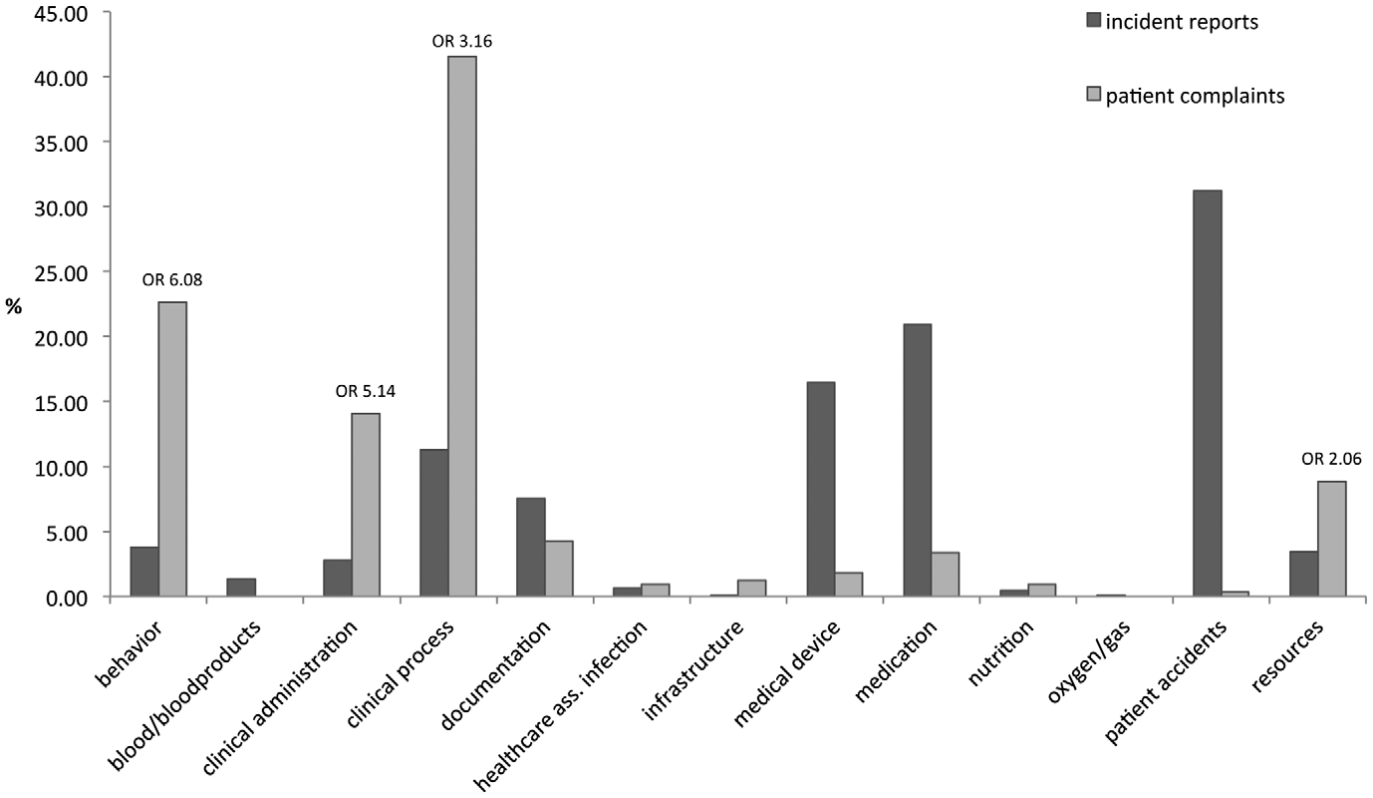
Distributions of incident reports and patient complaints over categories of the classifier ‘incident type’ (in %).

**Figure 2:**
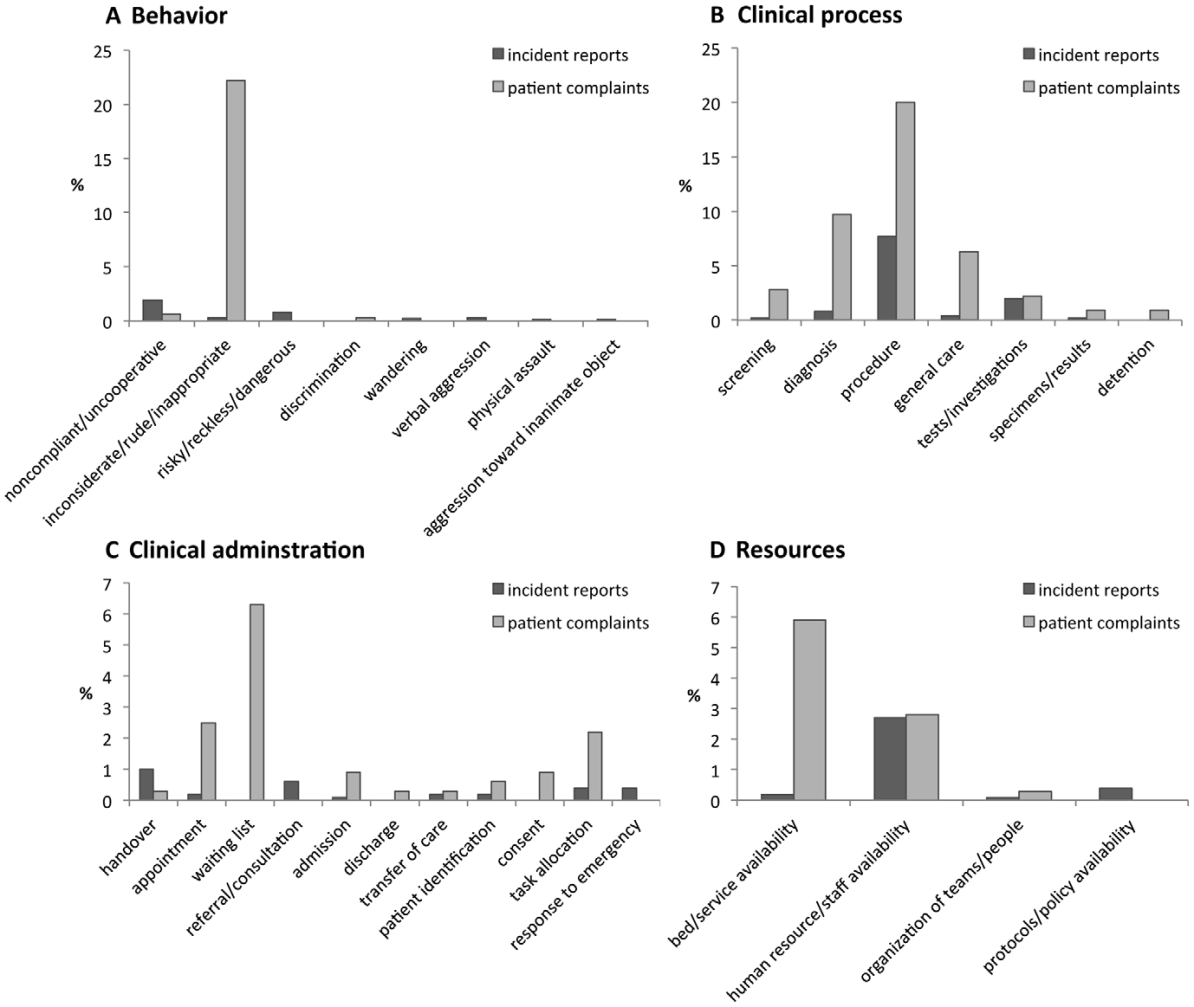
Distributions of incident reports and patient complaints over subcategories of Incident Types. A: subcategorie of “Behaviour”. B: subcategorie of “Clinial administration”. C: subcategorie of “Clinical Process”. D: subcategorie of “Resources/ organizational management”.

Figure 1 shows a substantial difference between the distributions of incident reports (IR) and patient complaints (PC), with a low PSI of 0.32. Some categories are strongly represented in PC and not in IR, and vice versa. The odds ratios show that incidents in the following four categories are more likely to be detected by patient complaints than by incident reports: behaviour (OR = 6.08), clinical administration (OR = 5.14), clinical process (OR = 3.16) and resources (OR = 2.06). The subcategories yield more detailed information about the differences. Figure 2a shows that the difference between the sources in the category behaviour relates primarily to inconsiderate/rude/inappropriate behaviour (0.3% (IR) vs. 22.2% (PC)). Differences in clinical administration (figure 2c) relate to appointments (0.2% (IR) vs. 2.5% (PC)), waiting list (0% (IR) vs. 6.3% (PC)) and task allocation (0% ( IR) vs. 2.2% (PC)). The difference in clinical process (figure 2b) relates to procedure (7.7% (IR) vs. 20.0% (PC)), diagnosis/assessment (0.8% (IR) vs. 9.7% (PC)) and general care (0.4% (IR) vs. 6.3% (PC)). Differences in resources (figure 2d) relate to bed/service availability (0.2% (IR) vs. 5.9% (PC)).

### Incident reports vs. charts of deceased patients

**Figure 3:**
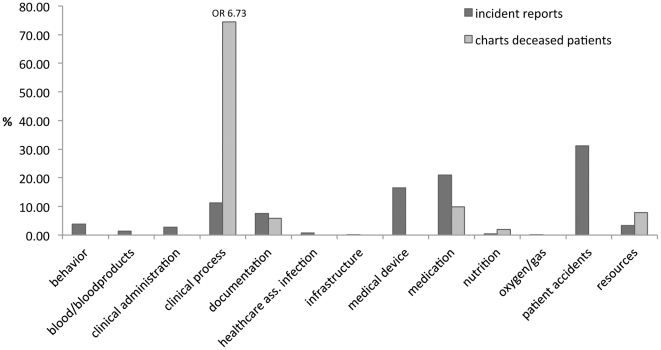
Distributions of incident reports and the chart reviews of deceased patients over categories of the classifier ‘incident type’ (in %).

**Figure 4:**
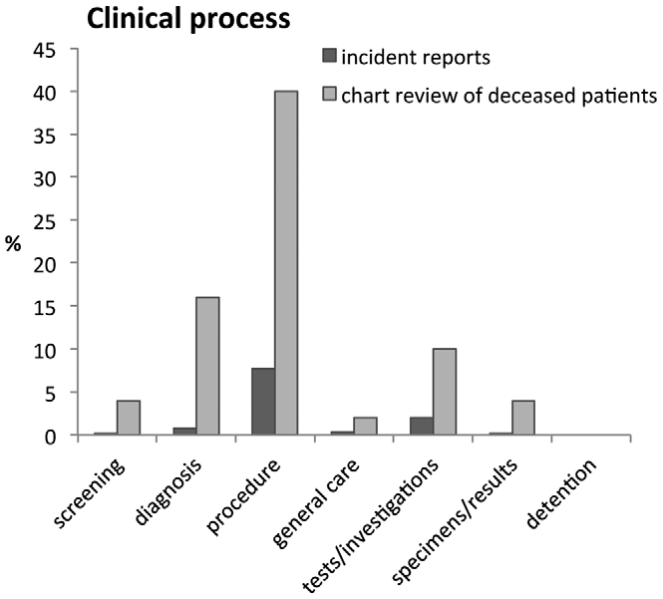
Distribution of incident reports and chart reviews of deceased patients over subcategories of “Clinical process”.

Figure 3 shows differences between incident reports (IR) and retrospective review of charts of deceased patients (CDP) (PSI 0.31) primarily related to clinical process (OR = 6.73). Figure 4 shows that this difference is mainly due to more reports from retrospective chart review relating to diagnosis/assessment (0.8% (IR) vs. 16% (CDP)) and procedure/treatment (7.7% (IR) vs. 40% (CDP)).

## Discussion

The primary aim of this study was to investigate whether information about reported incidents differed between information sources. The distribution of reports over categories and subcategories of the ICPS class ‘Incident Type’ showed remarkable differences between incident reports, patient complaints and retrospective chart review of deceased patients. This suggests that a combination of detection methods, using information from patients [14], [15], healthcare workers [3] and the gold standard of retrospective chart review [2], [8], may be preferable for studies of medical errors and patient safety in hospitals. Incident reports alone did not capture the full picture of medical errors, while other data sources, such as patient complaints and retrospective chart review, enhanced the comprehensiveness of information. The ICPS subcategories were particularly useful in specifying differences between information sources.

Patient complaints differed from IRS in several ways. First of all, patient complaints revealed more incidents in the category clinical process, particularly in relation to diagnosis, general care and procedure/treatment. Particularly striking is the difference between patient complaints and IRS in diagnosis-related incidents, mostly relating to delay in diagnosis or wrong or missed diagnoses. This is surprising, as one would expect healthcare workers to be aware of and therefore report missed diagnoses. The literature reports extensively on the prevalence of diagnostic errors and their impact on patient safety [35]–[38]. It is therefore intriguing that these errors should turn up in other types of data than IRS [35], [36], [38], [39] as was the case in our study. A possible explanation is that doctors may be aware of a wrong diagnosis, but decide that, since they know, there is no point in reporting it. Alternatively, doctors may not be aware of a wrong diagnosis, because it may take a long time before it is detected [35]. Whichever explanation applies, our study clearly shows that IRS do not suffice to reveal all diagnostic errors.

Secondly, patient complaints identified more incidents in the category behaviour, inconsiderate behaviour in particular. Previous research has shown that inconsiderate behaviour or unprofessional conduct is one of the main reasons for patient complaints or lawsuits [40]–[43]. In fact, it seems logical that this information should be found in patient complaints rather than in incident reports by hospital personnel, since the latter are unlikely to complain about their own behaviour.

Thirdly, patient complaints revealed more incidents in the category clinical administration in relation to waiting lists, management of appointments and task allocation, such as complaints about being seen or operated upon by a different doctor than expected or agreed upon. Complaints about waiting lists and management reports have also been reported elsewhere [40], [42]. They are closely related to patient complaints in the category resources, as patients tend to see insufficient resources as a cause for waiting lists, whereas doctors, who are familiar with the hospital organisation, know that delayed appointments or waiting lists cannot always be prevented due to staffing and organisational issues. It should be noted, however, that delays and problems with task allocation can cause significant harm to patients. A delay in treatment, for example, may lead to complications, while involvement of different doctors in a patient's treatment may cause handover problems, which are potentially harmful to patients [44].

Apart from patient complaints we gathered incident reports from retrospective chart review, which is generally considered the gold standard measurement of incidents occurring in hospitals [2], [8]. But even gold standards have limitations. For example, the fact that not everything is written down in charts, may lead to underestimation of the occurrence of incidents [7]. Our results show that retrospective chart review of inpatient deaths yields mostly incidents concerning delayed diagnosis and inadequate performance of procedures. With regard to diagnostic errors, the same applies for retrospective chart review as for patient complaints. These errors must be addressed in order to learn from them. As for inadequate performance of a procedure, incidents with medical procedures have also been identified in other studies involving retrospective chart review [9], [10].

### Limitations of this research

This study has several limitations. Firstly, most of the data were collected in one academic medical centre. Consequently, the results may not be generalisable to other hospitals or other countries. Secondly, because of anonymity of patient and staff information, overlap between incidents from different sources could not be detected. This might result in a slight overestimation of some incident types. Thirdly, we used ICPS to classify incidents in order to improve the comparability of findings. However, the ICPS is still under development and needs to be tested with more and different databases of other healthcare centres in order to optimise the (sub)categories.

### Practical implications and conclusions

There are also several practical implications to this study. First of all, the results suggest that IRS alone does not provide a comprehensive picture of what goes wrong in a hospital. Moreover, the fact that diagnostic errors and delay in treatment are rarely reported in IRS impacts on actions undertaken to remedy and prevent such incidents. Healthcare centres using more than one method of incident detection (e.g. methods relying on patients and health care workers as sources of information) should combine these data, preferably using the same classification for each source, in order to enhance comparability. This will give a better insight into the most prevalent latent and active errors, and can help to prioritise which of these problems should receive immediate attention and which are less urgent.

The second practical implication considers its use for medical education. The incidents that were identified can be used to educate medical students, residents and faculty about patient safety issues. Incidents can enhance awareness of vulnerabilities of hospital organisations and identify which situations are more conducive to error. Increased attention through education could increase doctors' awareness of these situations and, consequently, reduce the number of (e.g. diagnostic) errors. We therefore recommend that medical schools should incorporate this information in their courses on patient safety.

## Supporting Information

Box S1 **Example of an incident classified in more than one category.**
(DOC)
