# Supplementary material for: A Comprehensive Overview of Medical Error in Hospitals Using Incident-Reporting Systems, Patient Complaints and Chart Review of Inpatient Deaths
Source: PLoS One. 2012 Feb 16;7(2):e31125. doi: 10.1371/journal.pone.0031125 (PMC3281055; doi:10.1371/journal.pone.0031125)
Supplement: Box S1 — Example of an incident classified in more than one category. (DOC) [file pone.0031125.s001.doc]

**Box S1. Example of an incident classified in more than one category**

***Incident:*** The pain medication infusion pump of multiple patients was not working. It was agreed that someone from anaesthesiology would come to set up the infusion pump again, because nurses are not allowed to do this. However, no one from anaesthesiology would come, resulting in a delay in pain medication for these patients.

***Classified in three categories***

1) Medical device/ equipment, because the pump was malfunctioning; 2) medication/iv fluids, because the patient did not receive the medication in time; 3) behaviour, because of unwillingness of anaesthesiology staff to come and help.
